# Hazardous Object Detection by Using Kinect Sensor in a Handle-Type Electric Wheelchair

**DOI:** 10.3390/s17122936

**Published:** 2017-12-18

**Authors:** Jeyeon Kim, Takaaki Hasegawa, Yuta Sakamoto

**Affiliations:** 1 Department of Creative Engineering at National Institute of Technology, Tsuruoka College, Tsuruoka, Yamagata 997-8511, Japan; 2 Division of Mathematics, Electronics and Informatics, Graduate School of Science and Engineering, Saitama University, Saitama 338-8570, Japan; takaaki@hslab.ees.saitama-u.ac.jp; 3 Takaoka Toko Co., Ltd., Tokyo 110-0005, Japan; yutasakamoto@hslab.ees.saitama-u.ac.jp

**Keywords:** handle type electric wheelchair, mobility, elderly people, Kinect

## Abstract

To ensure the safety of a handle-type electric wheelchair (hereinafter, electric wheelchair), this paper describes the applicability of using a Kinect sensor. Ensuring the mobility of elderly people is a particularly important issue to be resolved. An electric wheelchair is useful as a means of transportation for elderly people. Considering that the users of electric wheelchairs are elderly people, it is important to ensure the safety of electric wheelchairs at night. To ensure the safety of an electric wheelchair at night, we constructed a hazardous object detection system using commercially available and inexpensive Kinect sensors and examined the applicability of the system. We examined warning timing with consideration to the cognition, judgment, and operation time of elderly people. Based on this, a hazardous object detection area was determined. Furthermore, the detection of static and dynamic hazardous objects was carried out at night and the results showed that the system was able to detect with high accuracy. We also conducted experiments related to dynamic hazardous object detection during daytime. From the above, it showed that the system could be applicable to ensuring the safety of the handle-type electric wheelchair.

## 1. Introduction

**Figure 1:**
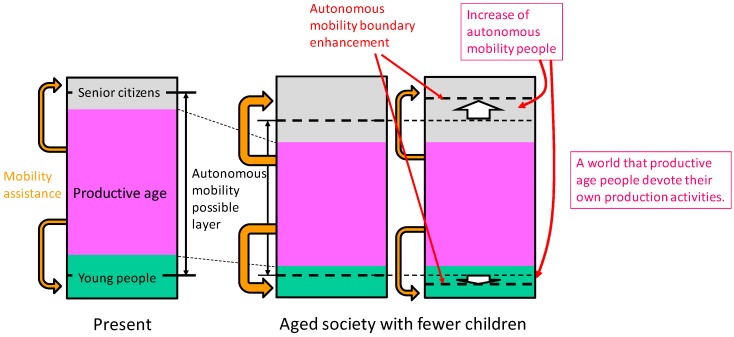
Ensuring the self-supportable mobility of elderly people and the vitality of society.

In a rapidly aging society, various social problems have arisen related to medical care, welfare, pensions, and ensuring the mobility of elderly people [1,2]. The latter is a particularly important issue to be resolved. Studies on ensuring the mobility of elderly people are actively conducted [3,4]. From the perspective of ensuring the vitality of an aging society as shown in Figure 1, it is important to ensure the autonomous (self-supportable) mobility of elderly people [5].

A handle-type electric wheelchair (hereinafter, electric wheelchair) is useful as a means of transportation for elderly people [6,7]. About 15,000 electric wheelchairs are sold each year in Japan [8]. The user of an electric wheelchair (maximum speed is 6 km/h) is a pedestrian under the Road Traffic Law in Japan. Elderly people use electric wheelchairs for shopping, hospital visits, participation in communities, walks, and so on. Truly, Reference [9] indicated that elderly people can achieve self-supportable mobility using electric wheelchairs, which underpins improvement in their quality of life (QoL). Nevertheless, falling accidents involving electric wheelchairs occur frequently on stairs, curbs, irrigation canals, and so on [10,11]. Many accidents occur during the daytime when elderly people move the most, but many accidents occur at night, when accidents can easily result in severe injury and death [11]. When elderly people go out during the day and come home late, night travelling is dangerous because the visibility of elderly people becomes worse at night. From these facts, it is an important task to ensure the safety of electric wheelchairs for use by elderly people.

To prevent falling accidents of electric wheelchairs, studies of hazardous object detection have been actively conducted [12,13,14,15,16,17,18,19,20,21]. Hazardous object detecting sensors in electric wheelchairs are classifiable into active type, passive type, and combined type. The active type sensors includes laser range finders, ultrasonic waves, and range image sensors (Time-Of-Flight type and Structured-Light type). The passive type sensors include stereovision and monocular cameras. Combined type sensors include a laser range finder and stereovision, monocular cameras and ultrasonic waves, and so on. Considering that electric wheelchair users are elderly people, safe driving support from evening to night (awareness enhancement) is an important subject. However, few reports describe hazardous object detection using commercially available and inexpensive equipment in electric wheelchairs.

To prevent falling accidents involving handle-type electric wheelchairs, we studied the applicability of commercially available and inexpensive Kinect sensors. Specifically, we constructed a hazardous object detection system using Kinect sensors, detected hazardous objects outdoors, and underscored the system effectiveness. The composition of this paper is the following. Section 2 presents a description of related research on hazardous object detection in electric wheelchairs. A hazardous object detection system using Kinect is proposed in Section 3. Furthermore, we evaluated the performance of static and dynamic hazardous object detection during nighttime, discussed in Section 4, and the performance of the dynamic hazardous object detection in the daytime is shown in Section 5. Finally, Section 6 presents conclusions.

## 2. Related Works

We describe related studies of hazardous object detection. Furthermore, ‘resolution’ in this paper means the ability to detect hazardous objects (height ± 0.05 m or more) within the hazardous object detection area described in Section 3. As explained earlier, hazardous object detecting sensors are classifiable into active type, passive type, and combined type.

First, the active type will be described. The active type includes laser range finders, ultrasonic waves, and range image sensors (Time-Of-Flight type, Structured-Light type). The laser range finder can acquire two-dimensional information by scanning with a laser. It is possible to detect a hazardous object with high accuracy during day or night on the scanning plane. In an earlier report of the relevant literature [12], the authors used a laser range finder to detect curbstones and to navigate. However, it is difficult to detect obstacles above and below the scanning plane. Moreover, these sensors are expensive. Time-Of-Flight (TOF) type and structured-light type sensors are range image sensors. The TOF type range image sensor irradiates light from a light source to an object, estimates the time until the reflected light returns for every pixel, and acquires three-dimensional information using the estimated time. Another report [13] describes obstacle detection by an indoor rescue robot. The TOF type range image sensor has resolution capable of detecting hazardous objects, but it is expensive because it estimates the time for each pixel. Additionally, it is difficult to use outdoors in the daytime when there is much infrared ray noise. The structured-light type sensor projects a specifically patterned light from the projector to the object. Then the pattern is photographed by a pair of cameras. The irradiation light pattern and the photographed light pattern are correlated [14]. Furthermore, three-dimensional information up to the object is obtained by triangulation as stereovision. Nevertheless, as with the TOF type, such sensors are difficult to use outdoors during daytime, when there is much infrared ray noise. Ultrasonic sensors estimate the distance using the time until the ultrasonic wave emitted from the sensor is reflected by the object. In one report from the relevant literature [15], navigation such as passing through a door and running along a wall can be performed using an ultrasonic sensor. Because ultrasonic sensors are inexpensive and compact, they are used frequently. They are useful day and night. However, because the resolution is low, it is difficult to detect small steps, which are hazardous objects for electric wheelchairs.

Next, passive type sensors include stereovision and monocular cameras. Stereovision acquires three-dimensional information by photographing the same object from two viewpoints. In an earlier report of the literature [16], obstacles and steps were detected using stereovision. Stereovision is inexpensive; moreover, the obtained image has high resolution. However, it is difficult to detect objects at night when the illumination environment is bad. Ulrich et al. used a monocular camera to detect obstacles indoors. The system converts color images to HSI (Hue, Saturation, and Intensity), creates histograms of reference regions, compares the surroundings with the reference region, and detects obstacles [17]. In a method using the monocular camera, calibration is easy because one camera is used. Mounting is easy because the size is small. However, it is difficult to use at night when the lighting environment is bad.

Finally, we describe a combined type using both active type and passive type for detecting hazardous objects. Murarka et al. used a laser range finder and stereovision to detect obstacles [18]. The laser range finder detects obstacles on the 2D plane while stereovision detects obstacles in 3D space, and a safety map is built. By combining the laser range finder and stereovision, the difficulty in detecting obstacles above and below the plane of the laser beam, which is a shortcoming of the laser range finder, was resolved. However, the laser range finder is expensive. As described in an earlier report of the relevant literature [19], 12 ultrasonic sensors and 2 monocular cameras were installed in an electric wheelchair to detect hazardous objects. Although sensors are inexpensive, it is difficult to use monocular cameras when the illumination environment is bad. Moreover, it is difficult to detect hazardous objects in electric wheelchairs because the ultrasonic sensor resolution is low. Furthermore, the necessity of using many sensors requires much time for installation and calibration of sensors.

Hazardous object detection using Kinect game sensors, which have multiple sensors in one device, will be described. In an earlier report from a study [20], the Kinect sensor was installed on a white cane for visually impaired people. Stairs and chairs are detected using range image data obtained from the range image sensor. Then, in another report [21], navigation is performed while avoiding obstacles using a laser scanner, ultrasound, and Kinect indoors. However, it takes time to install and calibrate the sensors because multiple sensors are used. Reference [22] examined obstacle detection (convex portion only) using Kinect v2 during the daytime and presented the possibility of obstacle detection by Kinect v2. However, no detailed explanation is available for the detectable range or the detection accuracy of obstacles by Kinect v2 during the daytime. Additionally, it is difficult to detect hazardous objects that are a concave area because the installation position (height) of Kinect v2 is low. When the installation position of Kinect is low, it might be estimated as higher than the actual height of the object. Furthermore, the system might not judge it as a hazardous object. It is difficult to detect without approaching the hazardous object.

To ensure the safety of electric wheelchairs using Kinect outdoors, it is necessary to detect static and dynamic hazardous objects using a commercially available and inexpensive sensor. However, few reports describe the performance evaluation of static (convex and concave portion) and dynamic (pedestrian) object detection using Kinect. As described in this paper, we assess the applicability of the commercially available and inexpensive Kinect sensor as a sensor for detecting hazardous objects to prevent falling accidents of electric wheelchairs.

## 3. Hazardous Object Detection System by Using Kinect Sensor

### 3.1. Hazardous Object Detection System

**Figure 2:**
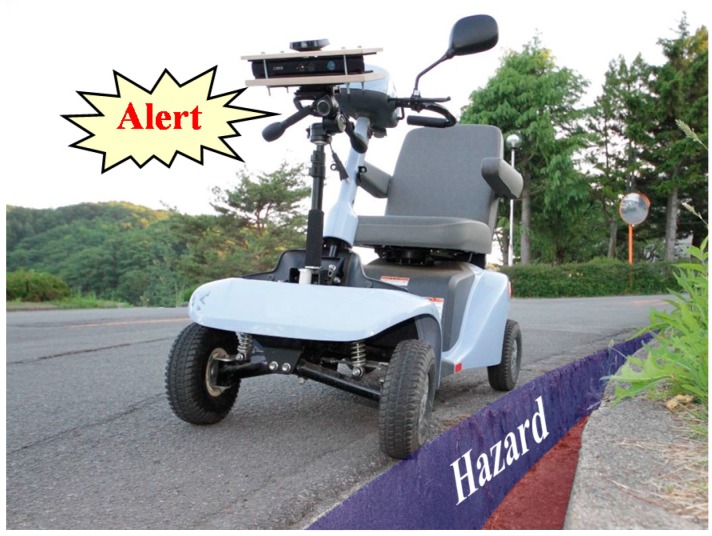
Image of the system.

A handle-type electric wheelchair, often used in Japan, is an effective candidate for use as a means of transportation for elderly people. To prevent falling accidents of handle-type electric wheelchairs on stairs, curbstones, irrigation canals, etc., the system presented in this paper detects static and dynamic hazardous objects using Kinect. The system does not control an electric wheelchair, but only presents warnings to users (elderly people) as presented in Figure 2. To detect hazardous objects, this system uses a Kinect range image sensor during nighttime and uses an RGB Kinect camera during the daytime. Then, the Kinect sensor is installed at a height (0.84 m) that does not disturb the user’s field of view. Five elderly people actually rode in the electric wheelchair. The heights of the five elderly people were 155–173 cm. A questionnaire survey was administered to the elderly person asking whether the Kinect sensor disturbs the front view or not. Based on the result, we obtained an answer that the view was not disturbed.

**Figure 3:**
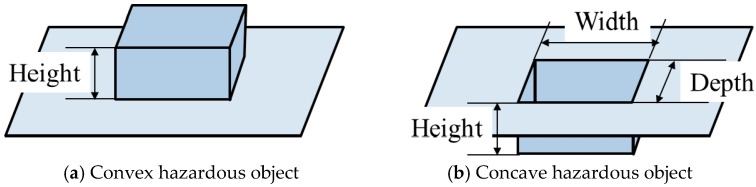
Static hazardous object.

Hazardous objects for electric wheelchairs include static and dynamic hazardous objects. Static hazardous objects, which do not move with respect to the surrounding environment, include curbstones, grooves, and irrigation canals. The static hazardous object size used in this study is decided based on Japan Industrial Standards (JIS) [5] and ISO 7176 [6] of the handle-type electric wheelchair (Figure 3). The convex portion of the static hazardous object is defined as an object with height of 0.05 m higher than the surroundings, as shown in Figure 3a. Then, the concave area of the static hazardous object is defined as an object for which all three of the following conditions are satisfied: height of −0.05 m or less, width of 0.10 m or more, and depth of 0.1 m or more, as portrayed in Figure 3b. In the case of the convex portion, the system judges it as a hazardous object when the difference between adjacent estimated values is +0.05 m or more. Moreover, in the case of a concave area, the system judges a hazardous object as one with object size satisfying all three conditions above (height −0.05 m or less, width 0.1 m or more, and depth 0.1 m or more). Furthermore, we assume that a dynamic hazardous object moves with respect to the surrounding environment and that it is in contact with the ground. Pedestrians, for example, are dynamic hazardous objects.

### 3.2. Hazardous Object Detection Area

Here, the warning timing for presenting a warning to the user (elderly person) is determined along with the hazardous object detection area. Time-to-Collision (TTC) [23,24] is used to assess the severity of traffic conflicts in this study. When traveling without changing the traveling direction at the current speed, TTC is the time until a collision with an object (hazardous object) or an accident occurs. Because the hazardous object detection system is aimed at preventing falling accidents, TTC is set as the severity of traffic conflicts in this study. Furthermore, the warning timing and the hazardous object detection area are decided using TTC.

The warning timing is a time when the system can give a warning to the user (elderly) and can stop safely. The warning timing is decided based on the recognition, judgment, and operation time of elderly people. The selection reaction time is used for the recognition and judgment time of elderly people. According to a report from an earlier study [25], because the average of the selection reaction time of an elderly person is 0.7 s and because the standard deviation is 0.13 s, 0.83 s is taken as the recognition and judgment time. When traveling for 0.83 s (recognition and judgment time) at the maximum speed (6 km/h) of the electric wheelchair, the traveling distance is about 1.38 m. The operation time is found based on the braking distance of the electric wheelchair, as defined in the JIS guideline [5]. According to JIS, the electric wheelchair must be able to come to a stop within 1.5 m on a flat road. The braking distance of many commercially available electric wheelchairs is within 1.2–1.3 m [10]. Traveling 1.5 m at the maximum speed (6 km/h) of the electric wheelchair takes 0.9 s, so the operation time is 0.9 s. The warning timing shall be 1.73 s from recognition, judgment, and operation time of elderly people. Setting the warning timing at 1.73 s when using the inexpensive Kinect can reduce the risk posed by electric wheelchairs.

Based on the results described above, the front side of the static hazardous object detection area is set to 3 m (2.88 m + margin 0.12 m). The width shall be 0.7 m, which is the electric wheelchair width according to JIS. The detection range of dynamic hazardous objects takes into account the relative speed of the electric wheelchair and dynamic hazardous objects.

## 4. Performance Evaluation in Nighttime

### 4.1. Hazardous Object Detection Method

We will describe the relative position acquisition method from the electric wheelchair to the object, measures against error caused by the change of road gradient conversion, and the process flow of the hazardous object detection.

**Figure 4:**
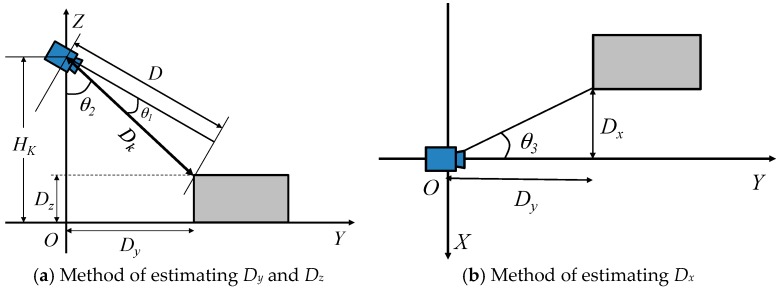
Method of estimating the relative position from the electric wheelchair to the hazardous object.

Figure 4 presents the estimation method of the relative position from the electric wheelchair to the object. Here, the height of the Kinect (*H_k_*) is known. The system obtains range image data (*D*) from the Kinect and calculates the distance (*D_k_*) from the Kinect to the object using Equation (1). Then, the system calculates *D_y_*, *D_z_*, and *D_x_* using Equations (2)–(4), respectively. Here, *D_x_* and *D_y_*, respectively, denote the distance in the *x* direction and the distance in the *y* direction from electric wheelchair to the object. Moreover, *D_z_* is the object height. When the object size meets the size of a hazardous object, as described in Section 3.1, it is judged as a hazardous object.

(1) $$D_{k}=D/\cos\theta_{1}$$

(2) $$D_{y}=D_{k}/\sin\theta_{2}$$

(3) $$D_{z}=H_{k}-D_{k}\cos\theta_{2}$$

(4) $$D_{x}=D_{y}\tan\theta_{3}$$

**Figure 5:**
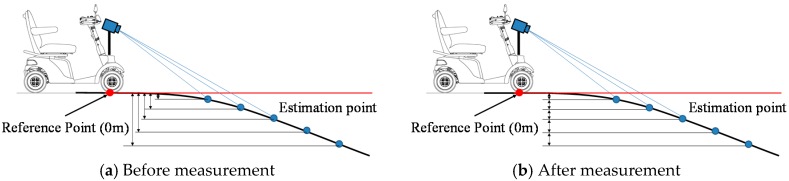
Measures against error caused by a change of road gradient conversion.

We now describe measures taken against error caused by the change of road gradient conversion. Here, the reference point (height 0 m) is just under the Kinect. Assume that there is a slight slope that the electric wheelchair can pass through, as shown in Figure 5. When the system judges hazardous objects using the difference between the height of the reference point and the height of the estimation point, the greater the distance between the reference point and the estimation point is, the larger the difference between the reference point and the estimation point becomes, as shown in Figure 5a. In spite of having a slope through which the electric wheelchair can pass, the system judges that it is a hazardous object (concave area). To resolve difficulties, the system judges a hazardous object using the height difference between adjacent estimation points, as shown in Figure 5b.

The process flow of hazardous object detection at night occurs as explained below. First, the system acquires the height information of estimation points in the hazardous object detection area using the range image obtained from the Kinect. Then the height difference between adjacent estimation points in each column direction of the range image is acquired. Next, when an estimation point with a height difference of +0.05 m or more is detected, the system judges the object as a convex hazardous object. Furthermore, when an estimation point with a height difference of −0.05 m or less is detected, a region where the depth of the concave portion is 0.1 m or more and the width is 0.1 m or more is detected. If all three conditions are satisfied, the system judges the object as a concave hazardous object. Finally, the position of the estimation point closest to the sensor in this region is inferred as the position of the hazardous object.

**Figure 6:**
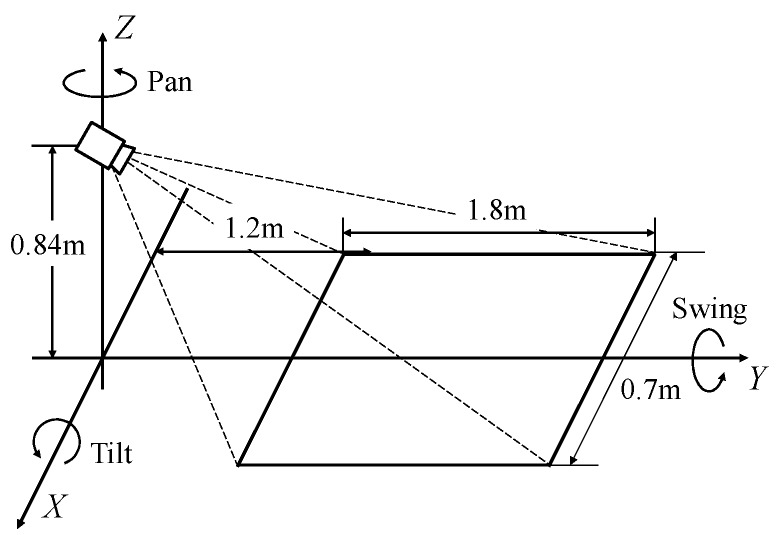
Installation of the Kinect, and the static hazardous object detection area.

Figure 6 shows the installation and the hazardous object detection area. The installed Kinect sensor height is 0.84 m. The Pan, Swing and Tilt of the installation angle are, respectively, 0 degrees, 0 degrees, and −26 degrees. The detection area of a static hazardous object by the installation angle and the angle of view of a Kinect sensor is from the sensor to 1.2–3.0 m as shown in Figure 6.

### 4.2. Experimental Method

**Figure 7:**
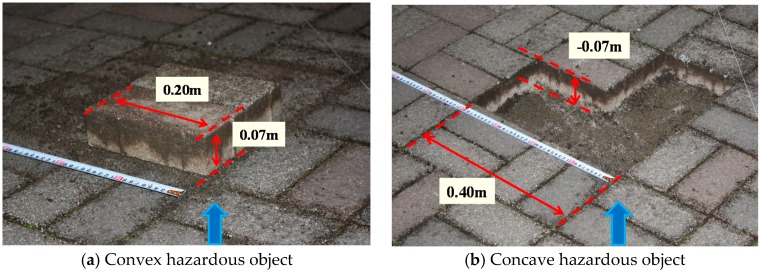
Static hazardous objects in experiments.

The electric wheelchair used for this study is an ET 4 E (Suzuki) [26]. The Kinect sensor (version 1) specifications are presented in Table 1. The static hazardous objects are formed as convex portions and concave portions of an interlocking block (Figure 7). The dynamic hazardous objects are pedestrians in this study.

**Figure 8:**
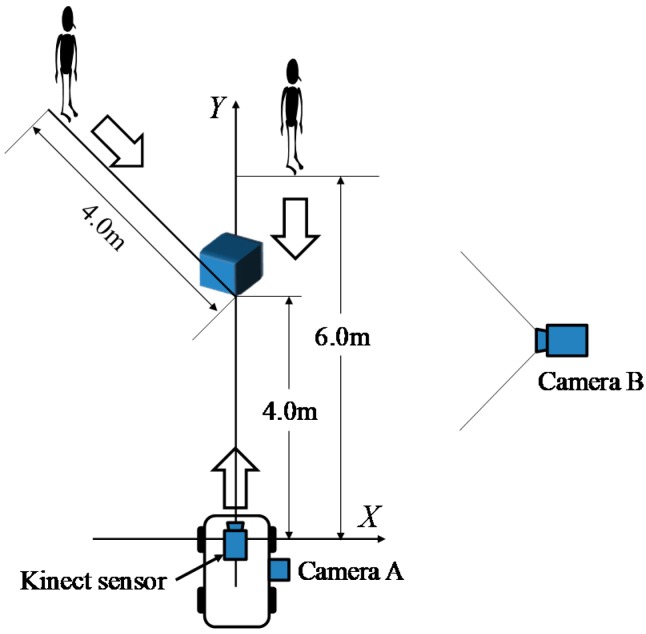
Experimental methodology.

The experimental method is shown in Figure 8. In the static hazardous object detection experiment, the electric wheelchair runs straight at 4 km/h toward the hazardous object (convex and concave hazardous objects) installed 4 m ahead from the start point. Additionally, a pedestrian–a dynamic hazardous object– approached from the position 6.0 m (front and diagonal 45 degrees). Then, the Kinect equipped on the electric wheelchair records range image data while moving. The system does not distinguish a static hazardous object and a dynamic hazardous object, and judges only whether or not it is a hazardous object in this paper. The performance evaluation is conducted by classifying cases of static and dynamic hazardous objects separately.

**Figure 9:**
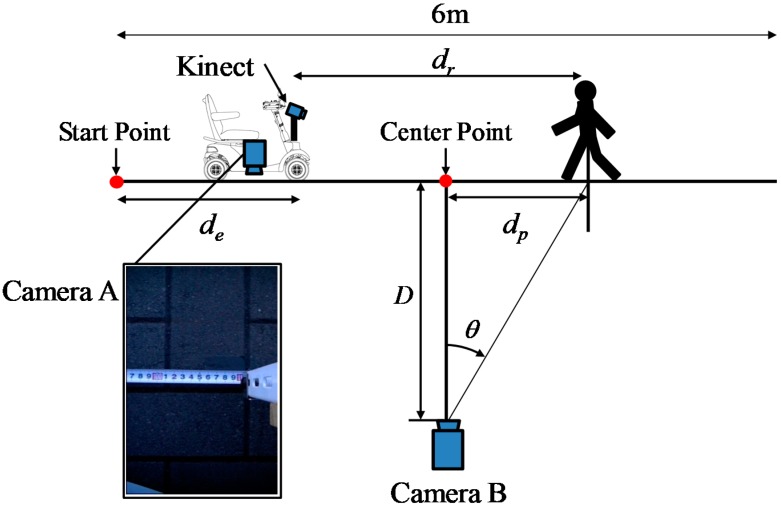
Method of acquiring the relative position of static and dynamic hazardous object from the electric wheelchair.

**Figure 10:**
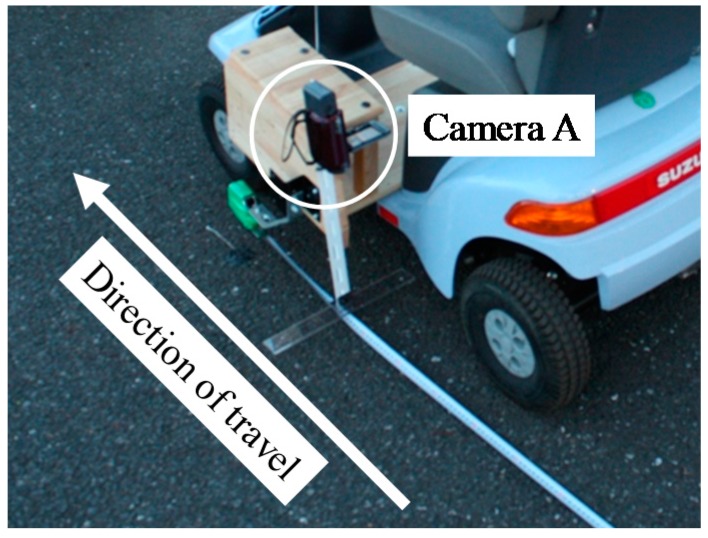
Installation of Camera A.

The method of obtaining the true value (position) of the electric wheelchair and the dynamic hazardous object (pedestrian) is now explained. For accurate performance evaluation of the hazardous detection system, it is necessary to acquire the precise positions of the electric wheelchair and the dynamic hazardous object. Because the static hazardous objects are fixed, the position of the static hazardous objects is not acquired. It is assumed that the electric wheelchair and the dynamic hazardous object move in a straight line. Furthermore, the true value of the dynamic hazardous object is defined as the torso position of the pedestrian. In cases of collisions between an electric wheelchair and a pedestrian, we defined that the criterion for determining whether or not an electric wheelchair and a pedestrian will contact is the position of the torso of the pedestrian. Figure 9 shows a method of acquiring the relative positions of the static and dynamic hazardous objects from an electric wheelchair. Camera A and Camera B are the true value acquisition cameras for performance evaluation experiments: Camera A is for the true value acquisition of electric wheelchairs, while Camera B is for the true value acquisition of the dynamic hazardous object. The Kinect is for performance evaluation experiments. Furthermore, Camera A and Camera B are time-synchronized with the Kinect. Camera A records a measuring tape on the road surface. In addition, the moving distance (*d_e_*) from the Start Point to the electric wheelchair of each frame from the video is obtained by visual observation. Then, the relative position from the electric wheelchair to the fixed static hazardous object is obtained. Figure 10 depicts the installation of Camera A. Next, the method of acquiring the relative position of the dynamic hazardous object (pedestrian) from the electric wheelchair is described. Acquisition of the position of the electric wheelchair is the same as described above. Camera B photographs the dynamic hazardous object, and the angle (*θ*) from the coordinates of the dynamic hazardous object on the image inputted from Camera B is then calculated. The position (*d_p_*) of the dynamic hazardous object from the Center Point is calculated using Equation (5). Then, the relative position (*d_r_*) from the electric wheelchair to the dynamic hazardous object is calculated using Equation (6).

(5) $$d_{p}=D\tan\theta$$

(6) $$d_{r}=\;6-d_{e}+d_{p}$$

Here, *D* is the distance from the Center Point to Camera B.

**Figure 11:**
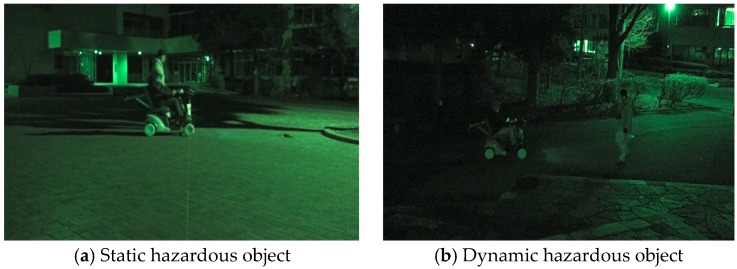
Experimental scene during nighttime.

The acquisition range of the position of the static hazardous object from the electric wheelchair is within 1.2 m in front of the electric wheelchair from 4.0 m. In the case of the dynamic hazardous object (pedestrian), it is from 1.2 m ahead to 6.0 m. In addition, the results obtained when the system estimates the relative position of the hazardous objects from the electric wheelchair are taken as estimated values. Five trials were used for each hazardous object experiment. The experiment location is Saitama University and experimental scenes during nighttime are shown in Figure 11.

The performance criteria are the detection rate and the estimation error in the hazardous object detection. Judgment of hazardous objects in the hazardous object detection area is done for each frame. In the case of the detection of a static hazardous object, if it is judged that there is no hazardous object because there are no hazardous objects within 3 m from the Kinect sensor up to the first 1 m after the departure of the electric wheelchair, we call it a True Negative. Moreover, if it is judged that there is a hazardous object in the remaining 3 m, as shown in Figure 8, we call it a True Positive. The detection rate (Accuracy) is the proportion of the sum of the number of frames of True Positive and True Negative to the total number of frames. Here, the Actual Positive is a case in which there is a hazardous object in the hazardous object detection area and the Actual Negative is a case in which there is no hazardous object in the hazardous object detection area. Here, the static hazardous object detection area is 1.2 m to 3.0 m, and the dynamic hazardous object detection area is 1.2 m to 4.0 m. The estimation error is the difference between the above-mentioned true value and the estimated value for each frame in the hazardous object detection area.

### 4.3. Results

First, we examined the validity of the warning timing before evaluating the system performance. Driving experiments were conducted with students because driving experiments with the participation of elderly people were difficult. Driving experiments, in which subjects stopped when an alarm sounded while driving at 6 km/h, were conducted. Results of 20 trial experiments demonstrated that all subjects were able to stop the electric wheelchair within 1.5 m.

Next, static hazardous object detection will be described. Table 2 and Table 3 present results of detection rates of convex hazardous objects and concave hazardous objects. The detection rate of convex hazardous objects was about 95% (516/541). The detection rate of the concave hazardous objects was also about 95% (514/540). The cause of false detection (False Negative and False Positive) was the estimation error that results from a change in the road surface slope and the vibration. These are the cases where the system judged that it was not within 3 m although the hazardous object was within 3 m, and where the system judged that it was within 3 m although the hazardous object was not within 3 m. It is noteworthy that all such false detections occurred when hazardous objects were more than 2.7 m distant from the electric wheelchair.

**Figure 12:**
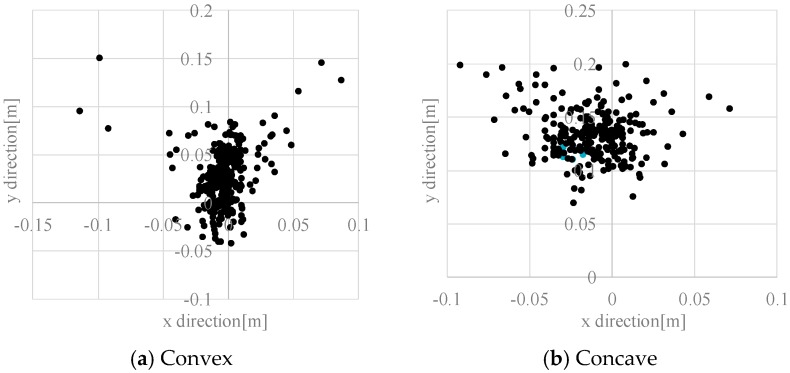
Distribution of estimation error of static hazardous object.

**Figure 13:**
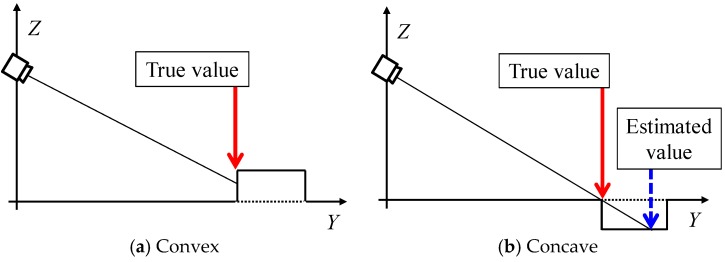
Reason of average error in the static hazardous object.

Figure 12 presents the distribution of estimation errors of convex and concave objects. The standard deviation was about 0.02–0.03 m. The cause of the error is estimation error because of the change of inclination of the road surface and vibration. In addition, the electric wheelchair cannot run straight while running. The average error of the *y* direction in convex objects is about 0.03 m, but the average error of the *y* direction in concave objects is +0.13 m. Figure 13 depicts the reason for the average error at the concave hazardous object. In the case of a convex portion, the estimated value is, in principle, the same place as the true value (the red solid arrow) because the estimated value is used when the system is judged to be a hazardous object, as shown in Figure 13a. However, in the case of a concave portion, the difference between the estimated value and the true value occurs because the estimated value (the blue dotted line arrow) is used when the system is judged to be a hazardous object as shown in Figure 13b. This is the main reason for generating the average error of the concave portion detection in the *y* direction.

**Figure 14:**
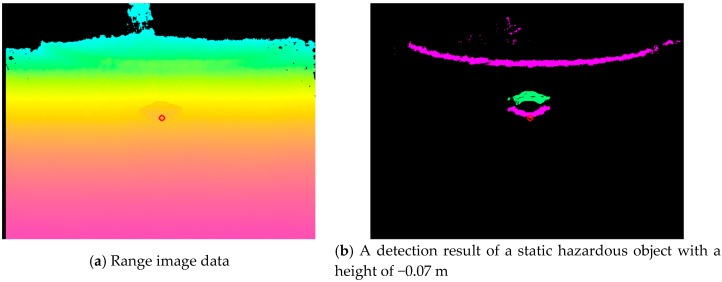
Example of the static hazardous object detection.

Finally, we describe experimentally obtained results of dynamic hazardous object detection. Table 4 and Table 5 present detection rates of dynamic hazardous objects in the front direction and the diagonal direction. The detection rates (Accuracy) in the front direction and diagonal direction were, respectively, 96% (356/370) and 97% (447/490). Figure 14 presents an example of detection of a static hazardous object. Figure 14a is the range image data and Figure 14b is the detection result of a static hazardous object with a height of −0.07 m. A white circle in Figure 14b means the closest place of the Kinect sensor. Here, sensor vibration is the main cause of False Positive and True Negative. Because of estimation error, the system determined that a hazardous object exists in the hazardous object detection area, even when there is no hazardous object in the hazardous object detection area.

**Figure 15:**
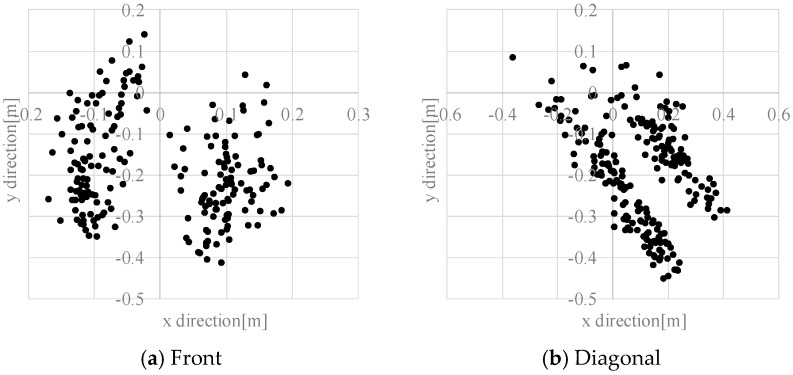
Estimation error in the dynamic hazardous object detection.

**Figure 16:**
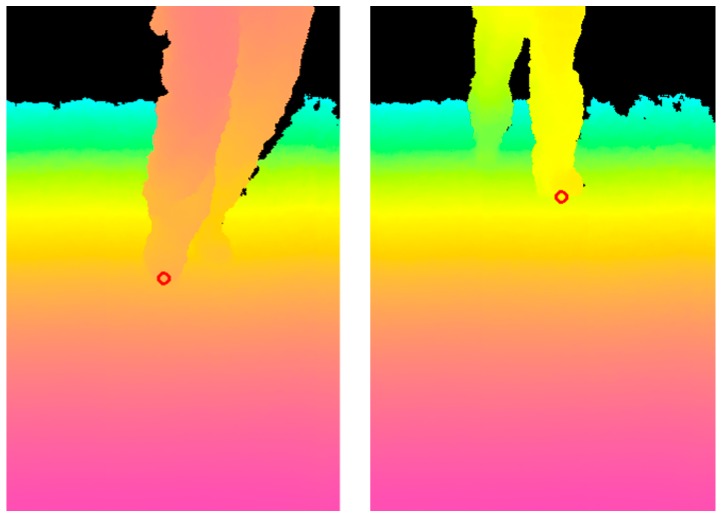
Example of the dynamic hazardous object detection.

**Figure 17:**
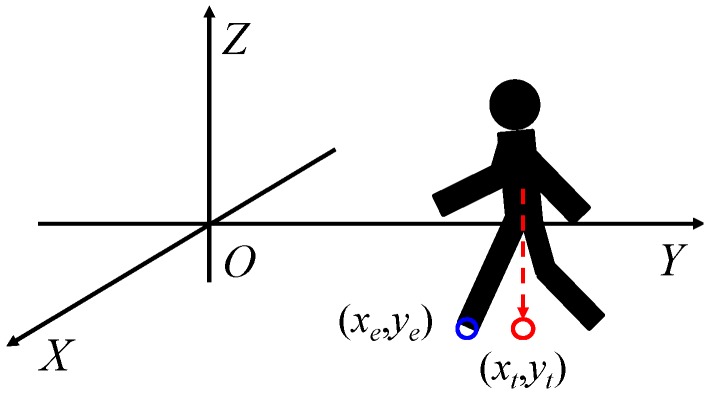
Reason of the average error in the dynamic hazardous object.

Figure 15 presents the distribution of estimation errors of a dynamic hazardous object in the front direction and the diagonal direction. The standard deviations were about 0.09–0.30 m. Figure 16 presents an example of the detection results of the left foot and the right foot. The distribution of the left foot and the distribution of the right foot are separated. The average error in the *y* direction is about −0.20 m, which means that the dynamic hazardous object was detected before the true position because the true position (*X_t_*, *Y_t_*) of the dynamic hazardous object is immediately under the torso in this experiment, but the actual detected place (*X_e_*, *Y_e_*) is the feet as shown in Figure 17. It also includes errors due to vibration during running.

**Figure 18:**
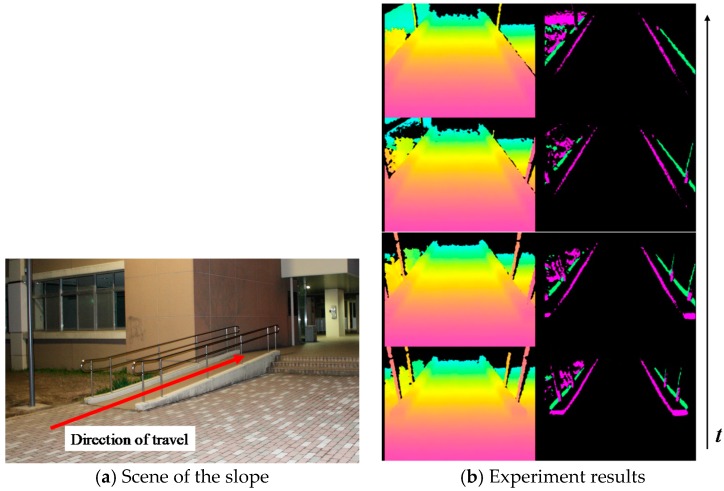
Results of the hazardous object detection in driving experiments on a slope.

Travelling experiments were conducted on a slope. One result is presented in Figure 18. Figure 18a portrays the scenery of the slope and travel direction given by the arrow. The left of Figure 18b depicts the inputted depth data from the Kinect. The right of Figure 18b displays the results of the hazardous object detection. The system distinguishes hazardous objects from the travelable slope. These results show that this system is effective for securing the electric wheelchair at night. However, it is necessary to perform the evaluation on detection of various hazardous objects under various environments.

## 5. Hazardous Object Detection in Daylight

### 5.1. Hazardous Object Detection Method

**Figure 19:**
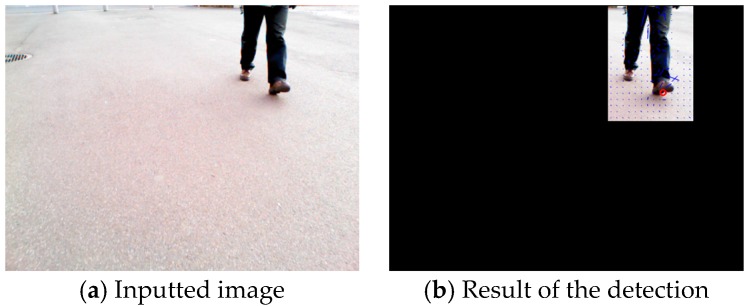
Example of the dynamic hazardous object detection by optical flow.

**Figure 20:**
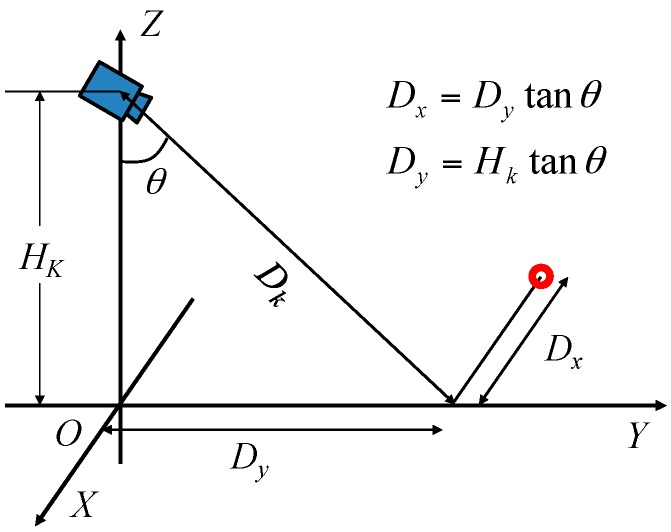
Method of estimating the position (*D_x_*, *D_y_*) of the dynamic hazardous object.

Dynamic hazardous object detection by optical flow during the daytime is now described. It is assumed that the dynamic hazardous object (pedestrian) is standing on the ground (0 m). The system obtains images from the Kinect RGB camera. Next, optical flow [27] is used to distinguish between the background and the dynamic hazardous object. The optical flow of the Point Of Interest (POI) is calculated using the gradient information of a total area of 75 pixels that are 5 pixels in the *x* axis direction, 5 pixels in the *y* axis direction, and 3 pixels in the time axis direction on the inputted image from the Kinect. The dilation–erosion process is used to remove noise. The area containing the dynamic hazardous object from the inputted image is cut out using optical flow. After performing binarization of the cut-out area, the edge of the dynamic hazardous object is extracted. Image coordinates of the lowest end of the edge of the extracted dynamic hazardous object are found. Figure 18 presents an example of the dynamic hazardous object detection by optical flow. Figure 19a is an inputted image. Figure 19b is a result of the detection. Finally, the obtained image coordinates are projected on the 0 m height plane (*x*–*y* plane) to estimate the position of the real-world coordinates. Then, the position (*D_x_*, *D_y_*) of the dynamic hazardous object is estimated as shown in Figure 20. Here, *H_k_* is the Kinect height, which is known.

### 5.2. Experimental Method

**Figure 21:**
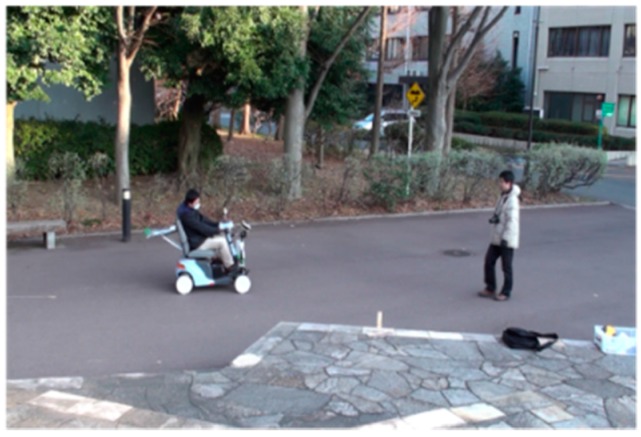
Experimental scene in daylight.

For each of five trials in the experiment, the pedestrian approaches from a position 6 m away (front and diagonal 45 degrees), as shown in Figure 8. The detection area of the dynamic hazardous object is 5.5 m ahead considering the relative speed of the electric wheelchair (maximum speed 6 km/h) and the pedestrian (4 km/h). Then, the Kinect equipped on the electric wheelchair records RGB images (daytime) while moving. Figure 21 portrays the scene of the dynamic hazardous object detection experiment during the daytime.

### 5.3. Results

The detection of dynamic hazardous objects during the daytime will now be described. The detection rates of dynamic hazardous objects are shown in Table 6 and Table 7. The detection rate in the front direction was 83% (354/424). The detection rate in the diagonal direction was 81% (403/498). Many False Negatives show that it is difficult to distinguish between the background and the dynamic object in a location that is distant from the electric wheelchair because the difference between the optical flow of the background and the optical flow of the dynamic object was small as a result of image blurring.

**Figure 22:**
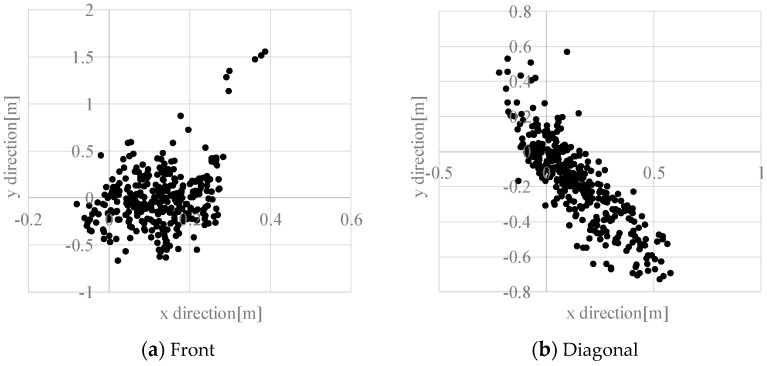
Distribution of measurement error in the dynamic hazardous object.

**Figure 23:**
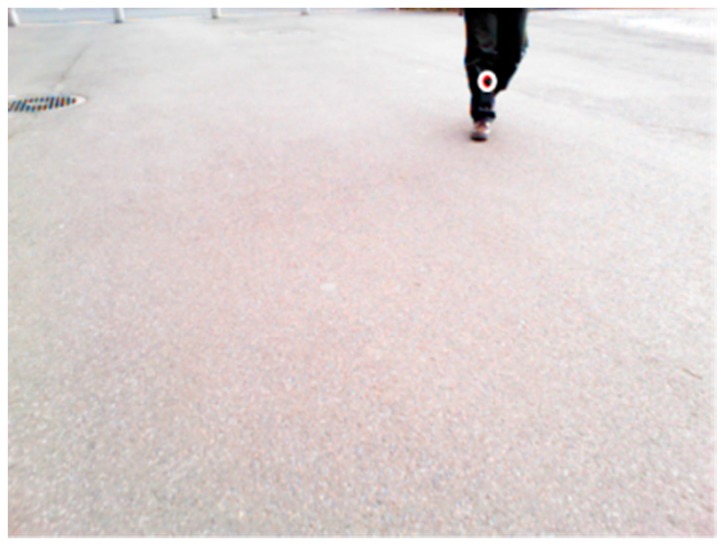
An example of dynamic hazardous object detection in daylight.

Estimation error related to dynamic hazardous object detection will be described next. Figure 22 depicts the distribution of the estimation error in the front direction and the diagonal direction. The standard deviations were about 0.09–0.30 m. The cause of the error is that the true value of the dynamic hazardous object is immediately under the torso, as explained in Figure 17, but the actual detected place is the foot. Furthermore, it is difficult to detect objects using the RGB camera of the Kinect during daytime because the Kinect RGB camera quality is wholly inadequate. The maximum error was 1.55 m. An especially large error in the estimation error results from detection of parts aside from the feet, as shown in Figure 23 (circles in the Figure). From these facts, it is necessary to improve the performance of the dynamic hazardous object detection during daytime.

## 6. Conclusions

This report has described the applicability of using a commercially available Kinect sensor to ensure the safety of a handle-type electric wheelchair. Considering that the actual users of electric wheelchairs are elderly people, it is important to ensure the safety of electric wheelchairs at night. To ensure the safety of the electric wheelchair during nighttime, we examined warning timing and the hazardous object detection area while considering the recognition, judgment, and operation time of elderly people and constructed a hazardous object detection system using Kinect sensors. The results of detecting static and dynamic hazardous objects outdoors demonstrated that this system is able to detect static and dynamic hazardous objects with high accuracy. We also conducted experiments related to dynamic hazardous object detection during the daytime. Along with the results presented above, it showed that this system is applicable to ensuring the safety of the handle-type electric wheelchair.

The results described above demonstrated that the system is useful for hazardous object detection with an electric wheelchair using a commercially available and inexpensive Kinect sensor. Furthermore, the system can reduce the risk associated with the use of electric wheelchairs and contribute to securing the autonomous mobility of elderly people.

As future tasks, further performance evaluations on the detection of various hazardous objects under various environments should be undertaken, because only the typical examples of convex and concave portions were evaluated for performance. Additionally, there should also be studies on the detection of the static hazardous objects during daytime and the improvement of dynamic hazardous object detection by tracking. Furthermore, a comparison with other methods of static hazardous object detection should be carried out.

## Figures and Tables

**Table 1:** Specifications of Kinect v1.

Model	Kinect Version 1
Resolution of RGB camera	640 × 480
Resolution of range image sensor	320 × 240
Range of range image sensor	0.8~4.0 m
Angle of view (horizon)	57 degrees
Angle of view (vertical)	43 degrees

**Table 2:** Detection rate of convex (nighttime).

	CP *	CN *
AP *	334	15
AN *	11	180

* AP, Actual Positive; AN, Actual Negative; CP, Classified Positive; CN, Classified Negative.

**Table 3:** Detection rate of concave (nighttime).

	CP *	CN *
AP *	324	25
AN *	0	192

* AP, Actual Positive; AN, Actual Negative; CP, Classified Positive; CN, Classified Negative.

**Table 4:** Detection rate of dynamic hazardous objects in the front direction (nighttime).

	CP	CN
AP	188	0
AN	14	168

**Table 5:** Detection rate of dynamic hazardous objects in the diagonal direction (nighttime).

	CP	CN
AP	224	5
AN	8	253

**Table 6:** Detection rate of dynamic hazardous objects in the front direction (daylight).

	CP	CN
AP	316	63
AN	7	38

**Table 7:** Detection rate of dynamic hazardous objects in the diagonal direction (daylight).

	CP	CN
AP	353	83
AN	12	50
